# A dual nozzle 3D printing system for super soft composite hydrogels

**DOI:** 10.1016/j.ohx.2021.e00176

**Published:** 2021-02-02

**Authors:** Andi Dine, Edward Bentley, Loic A PoulmarcK, Daniele Dini, Antonio E. Forte, Zhengchu Tan

**Affiliations:** a Department of Mechanical Engineering, Imperial College London, South Kensington Campus, Exhibition Road, London SW7 2AZ, UK; b John A. Paulson School of Engineering and Applied Sciences, Harvard University, Cambridge, MA 02138, USA; c Department of Electronics, Information and Bioengineering, Politecnico di Milano, Milan 20133, Italy

**Keywords:** Fused deposition, Additive manufacturing, 3D printing, Hydrogels, Flow rate control, Tissue scaffold fabrication

## Abstract

Due to their inability to sustain their own weight, 3D printing materials as soft as human tissues is challenging. Hereby we describe the development of an extrusion additive manufacturing (AM) machine able to 3D print super soft hydrogels with micro-scale precision. By designing and integrating new subsystems into a conventional extrusion-based 3D printer, we obtained hardware that encompasses a range of new capabilities. In particular, we integrated a heated dual nozzle extrusion system and a cooling platform in the new system. In addition, we altered the electronics and software of the 3D printer to ensure fully automatized procedures are delivered by the 3D printing device, and super-soft tissue mimicking parts are produced. With regards to the electronics, we added new devices to control the temperature of the extrusion system. As for the software, the firmware of the conventional 3D printer was changed and modified to allow for the flow rate control of the ink, thus eliminating overflows in sections of the printing path where the direction/speed changes sharply.

## Highlights

• An advanced 3D printing system for composite material tissue scaffold fabrication.
• Utilises a liquid-solid printing ink phase change for structural printing stability.
• The synergistic cryogel printing ink enables super soft 3D structures to be created.
• The dual nozzle extrusion system allows for the construction of complex architectures.

## Graphical abstract

**Figure:**
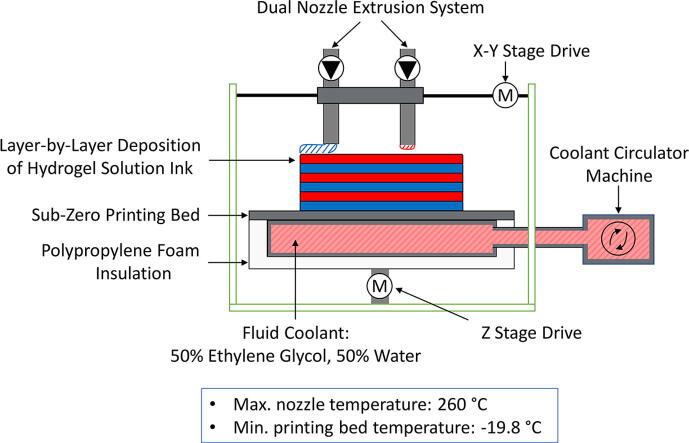

Specifications table

**Table:** 

Hardware name	*SSHDE 3D printer*
Subject area	• Engineering and Material Science • Chemistry and Biochemistry • Medical (e.g. Pharmaceutical Science) • Biological Sciences (e.g. Microbiology and Biochemistry)
Hardware type	• Biological sample handling and preparation • Electrical engineering and computer science • Mechanical engineering and materials science
Open Source License	CC BY-NC-SA 4.0
Cost of Hardware	*£1082.38 for additional modifications, £2873.37 including commercial 3D printer*
Source File Repository	*https://doi.org/10.5281/zenodo.3834063*

## 1 Hardware in context

Additive manufacturing (AM) by extrusion methods, widely known as three-dimensional (3D) printing and rapid prototyping (RP), is a manufacturing technique mainly used for the production of objects with complex geometries using layer-by-layer deposition techniques. The printing object is designed in digital form by means of computer-aided design (CAD) and translated into a consecutive stacking sequence of very thin layers of flowable material, which then becomes solid upon deposition. In recent years, medical research and the medical industry have taken on this promising technology and customized it with the aim of tackling new biological challenges [1].

Hydrogels are widely used in the medical field for their high water content [2] and very similar mechanical properties to those of human tissue [3], [4], [5], [6]. For these reasons they have potential use in the mimicking of organs that exhibit elastic moduli within the order of kPa [4], [6], [7]. The resultant artificial tissues can be used as training tools for surgeons [4], [6] and haptic virtual-reality simulators [8] due to their higher anatomical accuracy and lower cost when compared to animal models [4], [9], [10].

The shortage of organ donors is an additional factor that has driven the development of 3D printing techniques for tissue engineering applications. As an example, in 1999, Atala’s group 3D printed artificial bladders, which were implanted in human subjects [11]. This paved the way to an entire field of science, which nowadays focuses on the design of artificial human organs as a replacement for damaged ones. For example, 3D printing techniques are used to manufacture tissue scaffolds, often made by hydrogels [12] with the aim of mimicking the corresponding extracellular matrix. Cells, including stem cells, can then be seeded on the scaffold, which is then incubated in a bioreactor to foster cell maturation and proliferation [12]. Moreover, 3D printing techniques utilising cell encapsulated printing inks have given rise to the terms bioink and bioprinting [13].

Over the past decade, a significant amount of research has been conducted in the field of bioprinting, which lead to the design of manufacturing machines able to create medical devices for humans such as artificial bladders [11], polyetherketoneketone (PEKK) bone plates [14] and bioresorbable tracheal splints [15]. Hinton and co-workers presented a 3D printer capable of producing hydrogel structures (alginate, collagen and fibrin gels) within a gelatin thermo-reversible support bath, which was used to prevent the collapse of the hydrogel structure due to its own weight [16]. In 2002, Billiet and co-workers reviewed the advantages and limitations of scaffold-based and scaffold-free bioprinting, unravelling the importance of the hydrogel material in cell seeding [12]. In 2015, Ozbolat and Yu overviewed various bioprinting techniques and discussed the main challenges of fabricating *de novo* human organs for transplant [17]. More specifically, they divided bioprinting methods into laser-based writing, inkjet-based printing and extrusion-based deposition. In 2018, a low-cost syringe pump, compatible with open source 3D printers, was designed by Pusch and co-workers, advancing the development of extrusion-based bioprinters [18].

The concept of integrating a cryogenic step to 3D print hydrogels is relatively new and is also referred to as rapid freeze prototyping (RFP). The inception of this technique began with the use of water as a printing ink by Zhang, Leu and co-workers in 1999, where water was ejected to form solid ice structures [19], [20], [21]. Similarly, a 3D printer placed inside a fridge was developed at McGill University and is capable of building ice structures with complex geometry [22], and Biggs developed an ice cream 3D printer which uses liquid nitrogen (LN) and a freezer to solidify liquid cream [23]. In 2008, Pham and co-workers printed a chitosan lattice through layer-by-layer deposition inside a cryogenic chamber that was able to reach −45 °C [24]. Adamkiewicz and Rubinsky developed a different method to cryogenically 3D print hydrogels in which the object is printed directly in a liquid nitrogen (LN) bath [25]. In 2017, Tan et al. [26] developed a cryogenic 3D printer capable of producing super-soft 3D structures by freezing PVA-phytagel hydrogel solutions deposited on a cooled printing platform filled with solid carbon dioxide in an isopropanol bath.

The cryogenic technology is needed to solidify the hydrogel solution during the printing process, allowing the next layer to be deposited on top. When a specific type of hydrogel called a cryogel is used as the printing ink, for example poly-vinyl alcohol (PVA), physical crosslinks form during the freeze–thaw cycle [27], [28], [29]. Therefore, the cryogenic printing step works synergistically to crosslink the deposited material without using toxic chemical cross-linkers. Hence, when the printing ends, the frozen object is thawed, which allows physical crosslinks to form and leaves the hydrogel intact and ready to be used for tissue engineering applications.

In order to achieve a successful hydrogel print, there are several printing parameters that greatly affect the print quality, including the nozzle’s temperature and diameter, printing velocity and dispensing pressure [30], [31], [32]. Webb and Doyle [33] tried to optimize these printing parameters by developing the Parameter Optimization Index (POI) in order to achieve high print quality and minimum shear stress in the bioink. Similarly, Contessi Negrini et al. [34] investigated the optimization of the printing parameters when printing with methylcellulose-based hydrogels in scaffold-free applications. In this contribution, the main optimization study was conducted on the printing velocity to ensure an even layer height and low material overflow at the print corners.

This article reports the development of a 3D printer as a proof-of-concept prototype, capable of printing complex parts made of super-soft hydrogels. The system allows for fabrication of composite material tissue scaffolds, which can be used to investigate the effect of substrate anisotropy and directionality on cell behaviour and physiology. Additionally, it could also be used to create scaffolds that mimic a tumoral mass surrounded by healthy tissue. These complex structures cannot be created by traditional cast-moulding methods, and this contribution shows that AM provides a suitable solution. The printer was developed by designing and integrating new subsystems into a conventional 3D printer. In particular, a heated extruder, dual-nozzle extrusion system and cooling platform were developed. In addition, many changes were made to the electronics and software of the 3D printer. All hardware and software designs and alterations are detailed in the following sections, allowing researchers in various fields to easily replicate the machine and benefit from being able to 3D print geometrically complex tissue scaffolds made from materials that mimic the mechanical behaviour of biological tissues.

## 2 Mechanical design

The cryogenic 3D printer was formed by repurposing an existing conventional 3D printer, the Ultimaker 2 (Ultimaker, Netherlands), hence its price has been included in the total cost of the hardware, please see item 1 of the Bill of Materials. A conventional 3D printer was only used as a starting point for the development of an advanced cryogenic 3D printer so that the essential mechanical components shared by all printers, i.e. control of the xyz stage and extrusion motors, could be utilised without having to redesign these parts. Although the specific printer mentioned in this article is the Ultimaker 2, the same modifications and advancements can be implemented to any 3D printer that uses the bottom-up layer-by-layer extrusion printing method.

Some components were removed from the Ultimaker 2 due to space limitation and their redundancy in terms of functionality. The parts that were removed are the heating platform, the heater cartridge, the thermistor and the fans. The cryogenic 3D printer was developed by designing and integrating new subsystems (extrusion system, heating extruder and cooling platform) into the conventional Ultimaker 2 printer. All subsystems were designed parametrically in SolidWorks™ software (Dassault Systèmes, France) and manufactured using CNC milling and turning machines, a REPRAP custom built 3D printer, a hot wire machine and a laser cutter.

### 2.1 Extrusion system

**Fig. 1:**
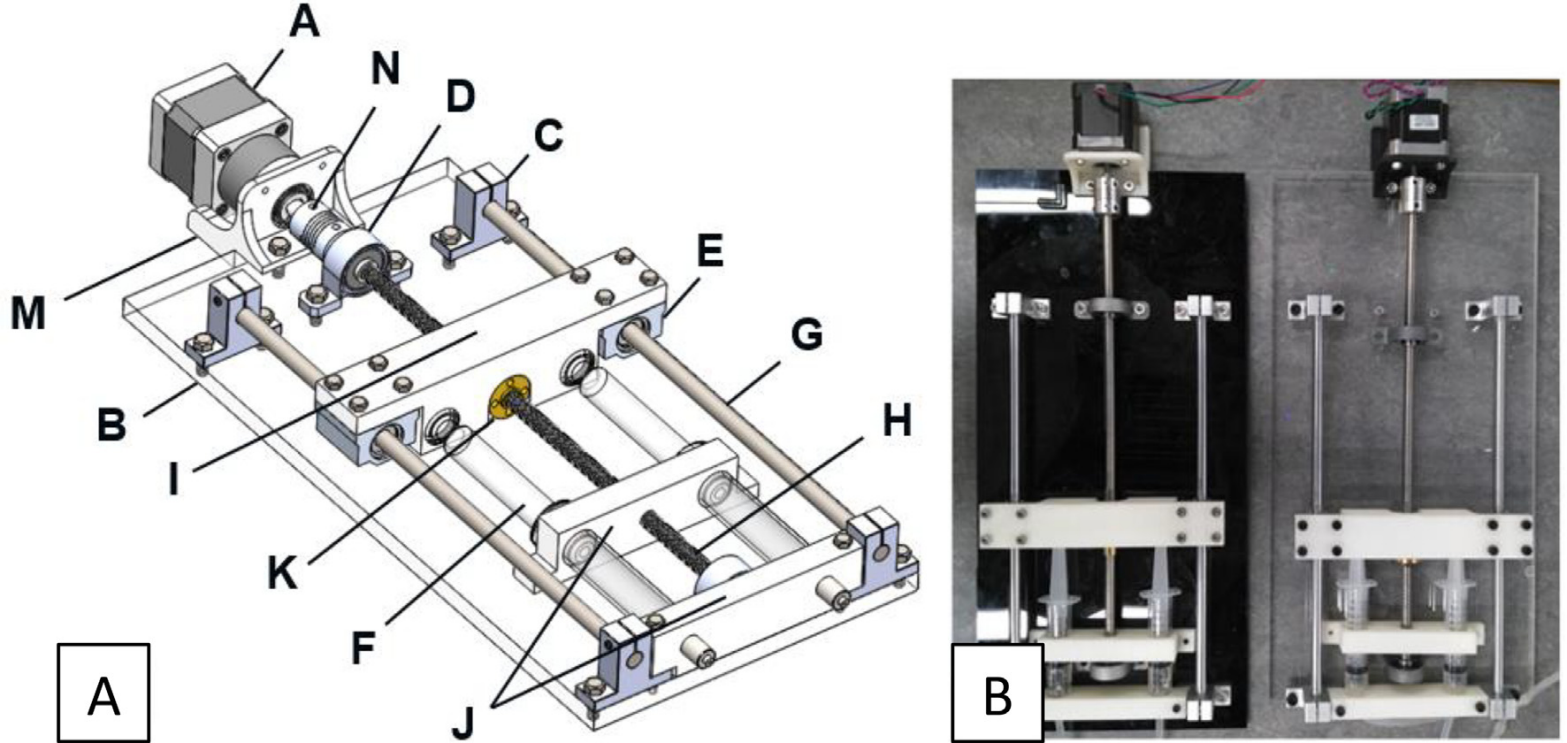
Extrusion system (A) designed using SolidWorks^TM^ software and (B) final construction of dual nozzle printing system using 10 ml plastic syringes.

The extrusion system consists of a stepper motor (A), a plexiglass block (B), four linear rail shaft supporters (C), two pillow bearings (D), two linear rails (E), two glassware syringes (F), two shaft rods (G), a lead screw (H), a coupler (N), two nuts (K) and some 3D printed parts (M, I and J see Fig. 1). The acrylic sheet acts as a base in order to keep all the parts at a fixed position. The four linear rail shaft supporters and the two pillow bearings are used to support the two shaft rods and the lead screw, respectively. The stepper motor is directly connected and controlled by the 3D printer and rotates the lead screw through a coupler. A 3D printed part (I) is connected to the lead screw through a nut in the middle, and also to two shaft rods through the linear rails at the edges. When the lead screw rotates, the rotation is transformed into a translation due to the shaft rods and the nut. Thus, part I pushes the plunger of the syringes to extrude the hydrogel. The rest of the 3D printed parts (J) support the syringes.

The extrusion system is adjustable, allowing the user to select the type of the syringes (glassware/plastic) as well as the capacity of them. More specifically, 10 mL glassware and 60 mL plastic syringes can be utilized by replacing the printed parts J.

### 2.2 Extruder head

**Fig. 2:**
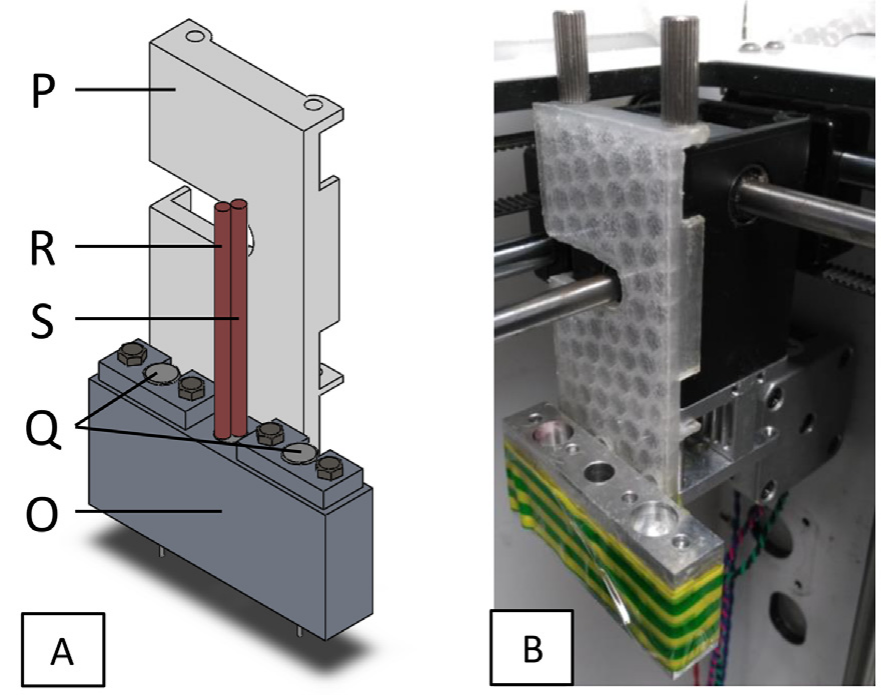
The extruder head (a) a fully assembled view using SolidWorks™ software and (b) after construction and attachment to Ultimaker XY moving stage.

The extruder head consists of a heating block (O) connected to the 3D printer’s extruder through a connector (P) (see Fig. 2). The connection between the heating block and the connector is achieved by four tight connections. The heating block was made from aluminium through subtractive manufacturing. The connector was made by 3D printing polylactic acid (PLA).

The heating block accommodates two needles (Q), a ceramic cartridge heater (R) and a thermistor temperature sensor (S), see Fig. 2. Needles of any gauge can easily be interchanged to allow for better control of printing inks of various viscosities; for example, water would require a higher gauge needle due to its lower viscosity compared to the PVA-phytagel hydrogel solution. In fact, the liquid-solid phase change of water to ice can be taken advantage of by using it as a supporting material, as previously demonstrated by Tan and co-workers [26]. This is because, after the freeze-thaw cycle, at room and physiological temperatures, the cryogel will remain intact whereas the supporting ice structure will simply melt away. Therefore, if any over-hanging print features are required, the use of purified water as a support material is recommended, as it is also non-toxic and biocompatible.

The ceramic cartridge heater is used to warm the heating block while the role of the temperature sensor is to feedback the temperature of the heating block to the microcontroller. The microcontroller used is an Arduino Mega2560 connected to a RAMPS 1.4 and to a full graphic controller (described in Section 3.4).

### 2.3 Cooling platform

**Fig. 3:**
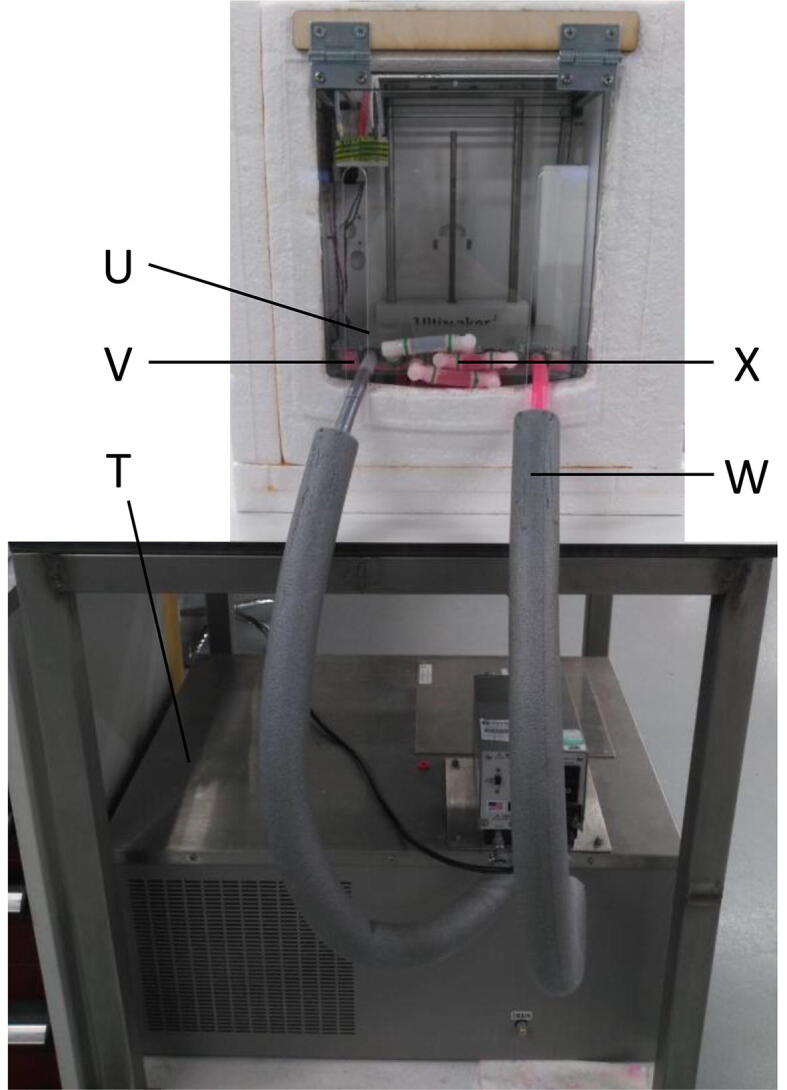
Cooling platform system overview showing polystyrene insulation surrounding printer.

For the cooling platform, one of the essential parts required to print super-soft hydrogels, a refrigerated circulating water bath machine (T, see Fig. 3) was used to freeze the printing platform through convection with antifreeze. More specifically, the total system consists of an aluminium plate (U) with four aluminium cooling blocks (V, attached below the aluminium plate to control the temperature of the printing platform and insulated with polypropylene), a VWR 1180-S refrigerated circulating water bath (T), a silicone tube with insulation protection (W), connectors and several jubilee hose clips (X). A complete deconstructed view and detailing the build of this system is provided in section 6.5. The temperature control of the printing platform is achieved by controlling the temperature of the antifreeze through the chiller which is then forced to pass through the cooling blocks.

The coolant [35] is a mixture of 50% ethylene glycol and 50% water, which has a freezing and boiling point of approximately −36 °C and 129 °C respectively. In addition, the coolant is based on organic acid technology providing corrosion inhibitors for the aluminium cooling blocks. The optimal temperature of the antifreeze was set at −25 °C while the flow rate of the antifreeze at 7.6 L/min. The cooling blocks were connected in series.

The tubes that connect the platform with the chiller are made by silicon and they have the following features [36]: hardness 60 (shore A), operational temperature from −60 °C to 200 °C, FDA and BFR approved. These characteristics make the tubes non-toxic and ideal for use in radiator and coolant systems. The tubes were covered with two layers of insulation protection to eliminate the unwanted heat transfer (V, see Fig. 3). Additionally, a thermal compound paste was applied to fill the air gaps between the aluminium platform and the heating blocks to increase the heat transfer. The connection between the cooling platform and the 3D printer is shown in Fig. 3.

**Fig. 4:**
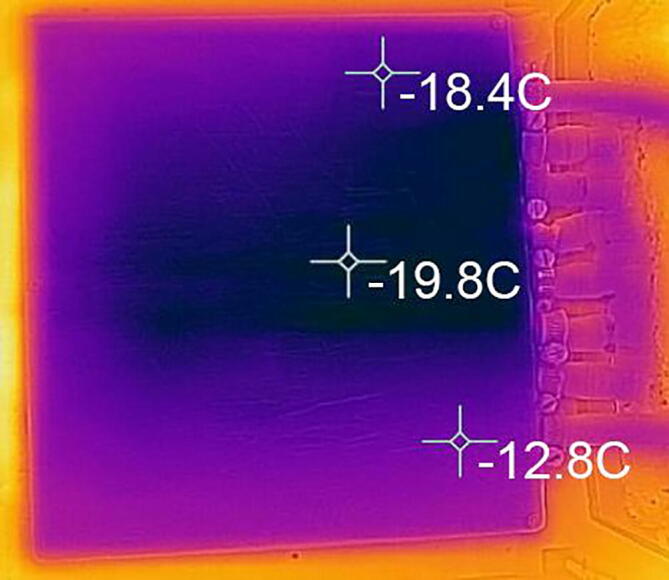
Temperature distribution over the printing platform.

The temperature distribution of the printing platform obtained with the coolant settings reported above is shown Fig. 4. The temperature distribution is quite uniform over the platform with the lowest achievable temperature reaching −19.8 °C and an upper limit of −12.8 °C. These temperature values are sufficient to consistently achieve good quality prints.

### 2.4 Thermally insulated chamber

The insulation chamber (see Fig. 3) reduces energy loss and allows the printer to achieve lower temperatures in a shorter timeframe. The insulation chamber was made from sheets of 50 mm expanded polystyrene surrounding the base and three sides of the 3D printer, excluding the top and front surfaces. All interfaces were sealed with either water-based adhesives, silicon sealants, or insulated tape. A hinged door made from acrylic was designed with two slits to allow enough space for the two protruding coolant tubes to move with the z-axis of the print platform.

## 3 Software and electronics

### 3.1 Firmware

As mentioned in Section 2, the basis of this cryogenic 3D printer is the Ultimaker 2 that was used mainly as an xyz movable axis system, which operates on its own firmware. However, the printer has a safety feedback control in order to detect the absence of any of these components and stop the printing operation. To avoid this, the Ultimaker firmware was changed to Marlin Firmware. This is open source software that allows the user to make changes in the code by using C programming language. The electronics that were no longer needed were disabled. Additionally, printing settings were changed (steps per unit length, initial performing height etc., please see following sections) in both the firmware and in the memory of the microcontroller, in order to achieve a good quality print.

Since the extrusion system has a limited range of rotations, the steps per unit length were decreased from 280 to 200 to reduce the rotation per step. By reducing the steps per unit length, we decreased the possibility of collisions. This parameter is saved in the EEPROM memory of the microcontroller for safety reasons and it was modified by executing the G code command M92. This command is stored in the machine settings of the 3D printer and executed by the printer at the beginning of any G-code program. Additionally, the steps per unit length were set equal to zero at the end of any G code program so as to eliminate the retraction motion when the printing ends.

### 3.2 Calculation of the deposited material

For the extrusion system a gauge 21 was chosen (although different gauges can be easily accommodated, and the following calculation can be adapted to cater for that). The needle has an outer and inner diameter equal to 0.82 and 0.51 mm, respectively. The cross-section of a single line of deposited material is assumed to be a circle with diameter equal to the inner diameter of the needle. The radial expansion of the hydrogel is assumed to be negligible compared to the main diameter of the extruded path [37], [38]. Therefore, the cross-section area, A, is equal to $0.204{\mathrm{mm}}^{2}$.

On average the printing speed of the hydrogel is 180 mm/min. Therefore, in one hour the total amount of hydrogel that is required is approximately $2448{\mathrm{mm}}^{3}$. The steps/unit length in the software were decreased from 280 to 200. By using this configuration, a full rotation of the extruder’s stepper motor is achieved when the extrusion command in G code is 21.5 steps/unit. The pitch of the T8 lead screw is 2 mm. Hence, one step in G code corresponds to ${9.3\times 10}^{-2}mm$ forward movement of the syringe’s plunger. The inner diameter of the 10 ml glassware syringe is 14.63 mm. Therefore, the extrusion volume of one step in the G code for two parallel 10 ml glassware syringes corresponds to 3126 mm^3^.

The required volume of hydrogel for a one-millimeter line is $0.204{\mathrm{mm}}^{3}$. Since one step in G code corresponds to 31.26${\mathrm{mm}}^{3}$, the steps for a one-millimetre line of hydrogel must be equal to 0.006526. It is worth recalling that these calculations are only valid for a gauge 21 nozzle.

### 3.3 Path programming

The path of the extruder was programmed by using the Slic3r software (https://slic3r.org/). The Slic3r is an open source 3D printer slicing application that generates the G code of a CAD model and allows the user to select the desirable printing parameters.

The diameter of the filament is the most important parameter since it determines the amount of the extruded material. More specifically, the amount of the deposited material (steps per millimeter) is proportional to the diameter squared. Firstly, the G code commands were generated through the Slic3r to specify the (default) steps of a one-millimeter line. The correct number of the steps per one-millimeter line was calculated in the previous Section 2.2. Therefore, one can estimate the multiplication factor for the diameter, if the correct and the default number of steps are known, as well as the relation between the steps and the diameter of the filament.

**Fig. 5:**
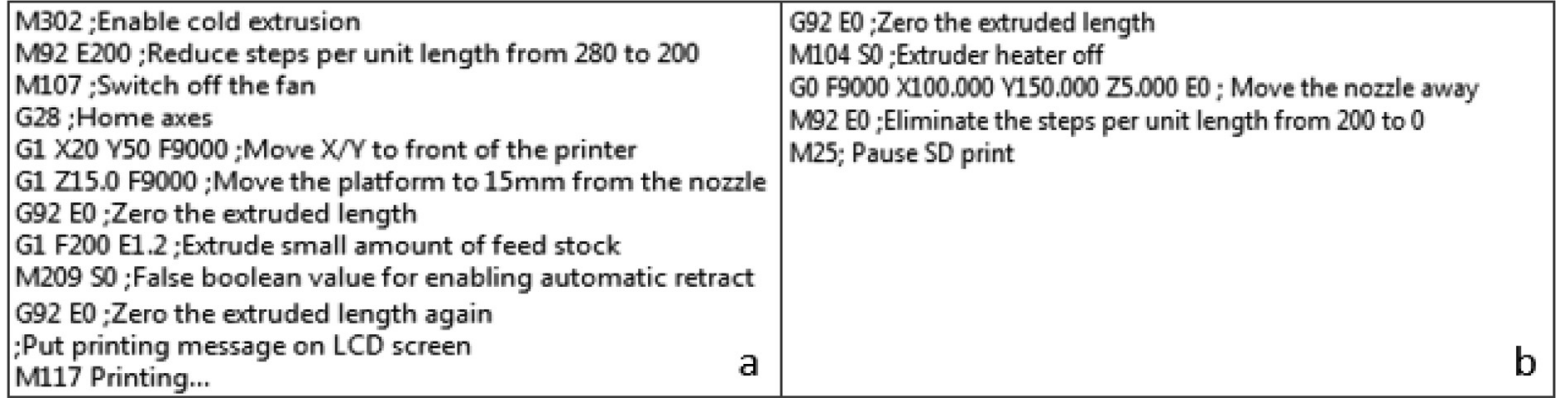
Custom G code a) At the beginning and b) at the end of any program file.

In addition, adjustments to the default Slic3r settings were made, including the activation of the “avoid crossing perimeters” and retraction activation. The nozzle diameter, extrusion width, overlap and extrusion multiplier were set equal to 0.5 mm, 0.55 mm, 0 and 1 respectively. The final width is slightly bigger compared to the real diameter of the nozzle due to the volume expansion of the hydrogel when frozen. Finally, the G-code flavour was defined as RepRap and a custom G code was set at the beginning and at the end of any program file to enable the change of the steps per unit length (see Fig. 5).

### 3.4 Heating system

**Fig. 6:**
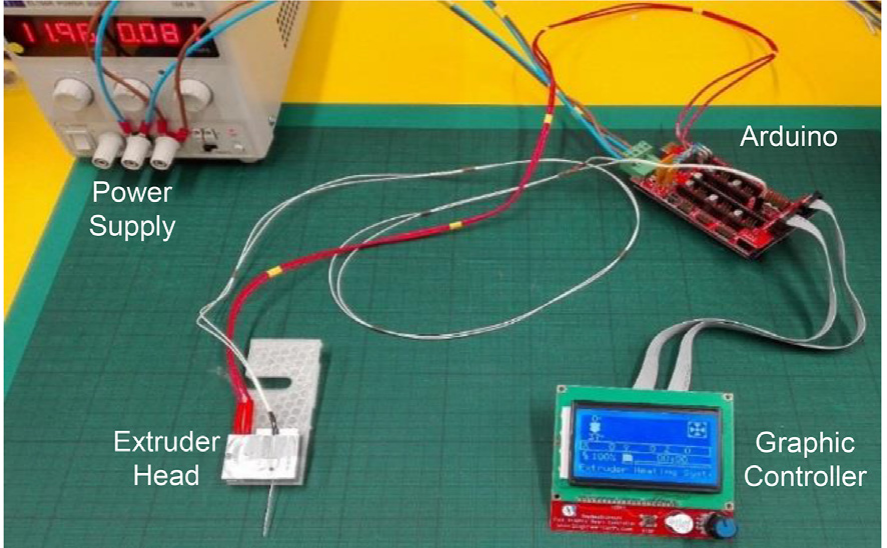
Extruder’s heating system. Close ups of the electrical connections are available in Section 5.4.

Marlin Firmware was chosen due to its simplicity and the low cost required electronics. The user can easily control the heating system by using a full graphic controller. The electronics for the heating system include an Arduino Mega2560, a RAMPS 1.4, a wired thermistor 100 kOhm (NTC), a heater cartridge cable (12 V – 100 W) and a workbench power supply (see Fig. 6).

When using the Marlin firmware, the software needs to be adjusted according to the applications. Most of the modifications to the Marlin Firmware were done in the folder Configuration.h of the Marlin. More specifically, an FGSC library for the full graphic controller was added, the communication speed (Baud rate) was set at 115,200 bps and all the electronics, the PID settings of the cartridge resistance as well as the safety configurations were specified as shown below:

• Full graphic controller & the SD card support were defined (#define ULTRA_LCD, #define DOGLCD, #define SDSUPPORT).
• Type of the motherboard: BOARD_AMPS_14_EFF.
• Power supply: ATX.
• Thermistor: 100 K NTC.
• PID of cartridge resistance: (DEFAULT_kp 36.96, DEFAULT_ki 2.61, DEFAULT_kd 130.79).

The preferable operation temperature of the heating block can be defined by the #PREHEAT_1_TEMP_HOTEND. Finally, the hysteresis time was increased to 2 min. This means that if the temperature stays constant or completely different from the target temperature for more than 2 min, the Marlin firmware will automatically switch off the printer as a safety precaution.

## 4 Design files

### 4.1 Design files summary

**Table:** 

Design file name	File type	Open source license	Location of the file
*Extruder_Head_Supporter (*Fig. 2*)*	*CAD file & STL file*	*CC BY-NC-SA 4.0*	*https://doi.org/10.5281/zenodo.3834063*
*Heating_Block (*Fig. 2*)*	*CAD file*	*CC BY-NC-SA 4.0*	*https://doi.org/10.5281/zenodo.3834063*
*Heater (*Fig. 2*)*	*CAD file*	*CC BY-NC-SA 4.0*	*https://doi.org/10.5281/zenodo.3834063*
*Needle_Holder (*Fig. 2*)*	*CAD file*	*CC BY-NC-SA 4.0*	*https://doi.org/10.5281/zenodo.3834063*
*M2_11 (*Fig. 2*)*	*CAD file*	*CC BY-NC-SA 4.0*	*https://doi.org/10.5281/zenodo.3834063*
*Needle (*Fig. 2*)*	*CAD file*	*CC BY-NC-SA 4.0*	*https://doi.org/10.5281/zenodo.3834063*
*Total_Assemby (*Fig. 2*)*	*CAD file*	*CC BY-NC-SA 4.0*	*https://doi.org/10.5281/zenodo.3834063*
*Bracket_Bearing (*Fig. 1*)*	*CAD file*	*CC BY-NC-SA 4.0*	*https://doi.org/10.5281/zenodo.3834063*
*Coupler (*Fig. 1*)*	*CAD file*	*CC BY-NC-SA 4.0*	*https://doi.org/10.5281/zenodo.3834063*
*D8 (*Fig. 1*)*	*CAD file*	*CC BY-NC-SA 4.0*	*https://doi.org/10.5281/zenodo.3834063*
*Glass Syringe Assembly (*Fig. 1*)*	*CAD file*	*CC BY-NC-SA 4.0*	*https://doi.org/10.5281/zenodo.3834063*
*Glass Syringe Barrel and Tip (*Fig. 1*)*	*CAD file*	*CC BY-NC-SA 4.0*	*https://doi.org/10.5281/zenodo.3834063*
*Glass Syringe Plunger (*Fig. 1*)*	*CAD file*	*CC BY-NC-SA 4.0*	*https://doi.org/10.5281/zenodo.3834063*
*Motor_Holder (*Fig. 1*)*	*CAD file & STL file*	*CC BY-NC-SA 4.0*	*https://doi.org/10.5281/zenodo.3834063*
*M4 (*Fig. 1*)*	*CAD file*	*CC BY-NC-SA 4.0*	*https://doi.org/10.5281/zenodo.3834063*
*M4_37 (*Fig. 1*)*	*CAD file*	*CC BY-NC-SA 4.0*	*https://doi.org/10.5281/zenodo.3834063*
*M4_165 (*Fig. 1*)*	*CAD file*	*CC BY-NC-SA 4.0*	*https://doi.org/10.5281/zenodo.3834063*
*M4_L20 (*Fig. 1*)*	*CAD file*	*CC BY-NC-SA 4.0*	*https://doi.org/10.5281/zenodo.3834063*
*M5_10 (*Fig. 1*)*	*CAD file*	*CC BY-NC-SA 4.0*	*https://doi.org/10.5281/zenodo.3834063*
*M5_14 (*Fig. 1*)*	*CAD file*	*CC BY-NC-SA 4.0*	*https://doi.org/10.5281/zenodo.3834063*
*M5_15 (*Fig. 1*)*	*CAD file*	*CC BY-NC-SA 4.0*	*https://doi.org/10.5281/zenodo.3834063*
*Motor_18 (*Fig. 1*)*	*CAD file*	*CC BY-NC-SA 4.0*	*https://doi.org/10.5281/zenodo.3834063*
*Nut_T8 (*Fig. 1*)*	*CAD file*	*CC BY-NC-SA 4.0*	*https://doi.org/10.5281/zenodo.3834063*
*Pillow_Bearing (*Fig. 1*)*	*CAD file*	*CC BY-NC-SA 4.0*	*https://doi.org/10.5281/zenodo.3834063*
*Plexiglass (*Fig. 1*)*	*CAD file*	*CC BY-NC-SA 4.0*	*https://doi.org/10.5281/zenodo.3834063*
*Pusher (*Fig. 1*)*	*CAD file & STL file*	*CC BY-NC-SA 4.0*	*https://doi.org/10.5281/zenodo.3834063*
*Shaft_Supporter (*Fig. 1*)*	*CAD file*	*CC BY-NC-SA 4.0*	*https://doi.org/10.5281/zenodo.3834063*
*Syringe_Supporter (*Fig. 1*)*	*CAD file & STL file*	*CC BY-NC-SA 4.0*	*https://doi.org/10.5281/zenodo.3834063*
*Syringe_Supporter_Back (*Fig. 1*)*	*CAD file & STL file*	*CC BY-NC-SA 4.0*	*https://doi.org/10.5281/zenodo.3834063*
*Total_System (*Fig. 1*)*	*CAD file*	*CC BY-NC-SA 4.0*	*https://doi.org/10.5281/zenodo.3834063*
*Tube (*Fig. 1*)*	*CAD file*	*CC BY-NC-SA 4.0*	*https://doi.org/10.5281/zenodo.3834063*
*Cryogenic 3D Printer Marlin Firmware*	*Compressed (zipped) Folder*	*CC BY-NC-SA 4.0*	*https://doi.org/10.5281/zenodo.3834063*
*Heating System Marlin Firmware*	*Compressed (zipped) Folder*	*CC BY-NC-SA 4.0*	*https://doi.org/10.5281/zenodo.3834063*

## 5 Bill of materials

All of the components listed in the bill of materials are standard components often already present in engineering workshops. It should be noted that the cost per unit and source of the materials mentioned in the bill of materials have been calculated for acquiring the exact quantity needed to build this hardware. However, these parts are usually acquired in bulk from engineering or scientific suppliers, such as RS Components or VWR International, thus we expect the total cost to build this machine to be lower for institutions with the appropriate faculties.

Bill of materials

**Table:** 

Designator	Component	Number	Cost per unit -currency	Total cost -currency	Source of materials	Material type
3D Printer (Fig. 3)	Ultimaker 2+	1	£1790.99	£1790.99	https://ultimaker.com/en/products/ultimaker-2-plus	Other
Extrusion system (Fig. 1)	Plexiglass	2	£9.59	£19.18	https://www.ebay.co.uk/itm/A4-Clear-Acrylic-Sheet-Perspex-Plexiglass-Plastic-Panels-210-x-297mm-Material/190703954331?epid=1293207251&hash=item2c66d7699b:m:mQqXRk4OJHl0oCO3aO_BE5Q	Acrylic
Extrusion system (Fig. 1)	Set: 400 mm Linear Shaft, Axi Rail Guide Shaft, Optical Bearing, Slide Support	2	£17.95	£35.90	https://www.ebay.co.uk/itm/15Pcs-400mm-Linear-Shaft-Optical-Axi-Rail-Guide-Shaft-Bearing-Slide-Support-UK/223516119330?hash=item340a995122:g:f8UAAOSwgR9c27oe&frcectupt=true	45 Steel
Extrusion system (Fig. 1)	M5x16mm Stainless Steel Allen Bolt	20	£0.13	£2.60 (10 pcs)	https://www.ebay.co.uk/itm/M4-M5-M6-M8-A2-STAINLESS-STEEL-ALLEN-BOLT-SOCKET-CAP-SCREWS-HEX-HEAD-DIN-912/221307549544?hash=item3386f53768:m:msfPgqjRcb8g1yvBmjFrAUw	Stainless Steel
Extrusion system (Fig. 1)	M4x40mm Stainless Steel Allen Bolt	4	£0.25	£1.25 (5 pcs)	https://www.ebay.co.uk/itm/M4-M5-M6-M8-A2-STAINLESS-STEEL-ALLEN-BOLT-SOCKET-CAP-SCREWS-HEX-HEAD-DIN-912/221307549544?hash=item3386f53768:m:msfPgqjRcb8g1yvBmjFrAUw	Stainless Steel
Extrusion system (Fig. 1)	M4x20mm Stainless Steel Allen Bolt	16	£0.135	£2.16	https://www.ebay.co.uk/itm/M4-M5-M6-M8-A2-STAINLESS-STEEL-ALLEN-BOLT-SOCKET-CAP-SCREWS-HEX-HEAD-DIN-912/221307549544?hash=item3386f53768:m:msfPgqjRcb8g1yvBmjFrAUw	Stainless Steel
Extrusion system (Fig. 1)	Syringes Luer Lock 60 ml	4	£4.50	£18.00	https://www.ebay.co.uk/itm/RAYS-Sterile-Syringes-Luer-Lock-60ml-30ml-20ml-10ml-5ml-3ml-1ml-NHS-UK-CE/182659772407?hash=item2a875eeff7:m:mpkZB1dU5O38H_F02yZ0asQ	Plastic
Extrusion system (Fig. 1)	Syringe 10ML	4	£5.64	£22.56	https://www.ebay.co.uk/itm/Glass-Syringe-Luer-Lock-Head-Glass-Injector-Syringe-Lab-Glassware-1ml-to-100ml/122103124893?var=421180158638&hash=item1c6de9d39d:m:mHPZMSx7X2s4GqUPadttgMw	Glass
Extrusion system (Fig. 1)	Flexible Shaft Coupling Coupler	2	£3.49	£6.98	https://www.ebay.co.uk/itm/Flexible-Shaft-Coupling-Coupler-Stepper-Motor-CNC-3D-Printer-4–5-6–35-8–10mm/283138679258?hash=item41ec6161da:m:mnK1i4L6w_6Xu1p2bLJkJSA&frcectupt=true	Aluminium
Extrusion system (Fig. 1)	Tubing Silicone 3 mm / 1 mm Wall > 200c	4 m	£1.50/m	£6.00	https://www.ebay.co.uk/itm/Silikonschlauch-Meterware-lebensmittelecht-Silicon-Schlauch-ab1mm-Innen/112607757467?hash=item1a37f2049b:m:mqvAS-Uq6_vdRxyCPJoAs2w&frcectupt=true	Silicone
Extrusion system (Fig. 1)	Connector Y-Piece 5 mm	2	£1.55	£3.10	https://www.ebay.co.uk/itm/Hose-Joiner-Plastic-Barbed-Connector-Pipe-Tubing-Fitting-Air-Fuel-Water-Vacuum/122046264580?hash=item1c6a863504:m:mm5Ycpp7p0RfYUc5d5N3FIA&frcectupt=true	Polypropylene
Extruder Head (Fig. 2)	Needle, Luer Lock 21gauge × 50 mm Stainless Steel	2	£2.79	£5.58	https://www.ebay.co.uk/itm/Stainless-steel-Luer-Lock-Blunt-Tip-Needles-Dispensing-Syringe-1–10Ga-30Ga-UK/253615779757?hash=item3b0cadafad:m:mTO3buFcFmzFovt2xW9HF1w&frcectupt=true	Stainless Steel
Extruder Head (Fig. 2)	M2 Stainless Steel Allen Bolt	4	£0.23	£1.15 (5 pcs)	https://www.ebay.co.uk/itm/M1-6-M2-M2-5-M3-A2-STAINLESS-STEEL-ALLEN-BOLT-SOCKET-CAP-SCREWS-HEX-HEAD-DIN-912/221306240232?hash=item3386e13ce8:m:msfPgqjRcb8g1yvBmjFrAUw&frcectupt=true	Stainless Steel
Extruder Head (Fig. 2)	Aluminium Block 60 mm × 27.5 mm × 15 mm	1	£9.00	£9.00	https://www.ebay.co.uk/itm/ALUMINIUM-BAR-BILLET-BLOCK-60mm-x-60mm-x-30mm-GRADE-6082-T6/264302917148?hash=item3d89ae761c:g:U-kAAOSw9VZXPEd2	Aluminium
Heating System (Fig. 6)	Set: USB RAMPS 1.4 + Mega2560 + 12864 LCD + A4988 Driver For 3D Printer Arduino Reprap	1	£26.31	£26.31	https://www.ebay.co.uk/itm/USB-RAMPS-1–4-Mega2560-12864-LCD-A4988-Driver-For-3D-Printer-Arduino-Reprap/153420274594?hash=item23b88f9ba2:m:m_KwFSRIaKBz3Sr_BruxBpA	Other
Heating System (Fig. 6)	Heater Cartridge	1	£0.99	£0.99	https://www.ebay.co.uk/itm/12V-24V-30W-40W-Ceramic-Cartridge-Heater-for-Arduino-3D-Printer-Heating-Element/272428429593?hash=item3f6dfff119:m:mtfn5kbXpDp4EoQi8o7DUXQ	Other
Heating System (Fig. 6)	Thermistor 100Kohm	1	£1.38	£1.38	https://www.ebay.co.uk/itm/Blesiya-1-100Kohm-NTC-Thermistors-Temperture-Sensor-for-3D-printer/253782329256?hash=item3b169b07a8:g:yIAAAOSw8KJb5H34	Other
Heating System (Fig. 6)	12 V AC/DC Volt Converter Regulated Switch Power Supply	1	£19.48	£19.48	https://www.ebay.co.uk/itm/12V-24V-5V-30A-AC-DC-Volt-Converter-Regulated-Switch-Power-Supply-for-LED-CCTV/382095146211?hash=item58f6a53ce3:m:m3p9ukC-hJu0KAr5fLBiECw	Other
Cooling Platform (Fig. 3)	VWR Scientific Bath/Circulator	1	£800	£800	https://www.capovani.com/iinfo.cfm?itemno=181977	Other
Cooling Platform (Fig. 3)	2-Way DIY Liquid Water Cooler Heat Sink Radiator 40*200 mm	4	£7.5	£30	https://www.ebay.co.uk/itm/2-Way-DIY-Liquid-Water-Cooler-Heat-Sink-for-CPU-Graphics-Radiator-40–200mm/362499247260?hash=item5466a38c9c:g:vRkAAOSwG1JcAmgN	Aluminium
Cooling Platform (Fig. 3)	XSPC PETG Tubing 14/10 mm	3 m	£7.68	£23.04	https://www.ebay.co.uk/itm/XSPC-PETG-Tubing-14–10mm-1m-Clear/162991399238?epid=1378191840&hash=item25f30b6146:g:U10AAOSwbpVbhrcb	Polyethylene Terephthalate Glycol
Cooling Platform (Fig. 3)	Barbed Elbow Tubing Connectors	6	£2.69	£16.14	https://www.amazon.co.uk/DEGREE-TEFEN-PLASTIC-BARBED-CONNECTOR/dp/B009XT6AV2/ref=sr_1_6?keywords=plastic+tubing+elbow&qid=1576448708&sr=8-6	Nylon
Cooling Platform (Fig. 3)	Jubilee Type Stainless Steel Hose Clamps Clips 8 mm-16 mm	10	£0.507	£5.07	https://www.ebay.co.uk/itm/PRIMA-jubilee-type-stainless-steel-hose-clamps-clips-6mm-10mm-up-to-200mm-220mm/121179363544?hash=item1c36da58d8:m:ms21-MU_6o3EAR9SMLSXd8w	Stainless Steel
Cooling Platform (Fig. 3)	Aluminium Sheet 500x250x2mm	1	£6.74	£6.74	https://www.ebay.co.uk/itm/Aluminium-Sheet-1mm-1-2mm-1-5mm-2mm-3mm-4mm-1050H14-S1BH4-Various-Sizes/271210657802?var=570160092864&hash=item3f256a380a:m:m2Gd-MD2AUW6CPxAPo4xh7Q	Aluminium
Cooling Platform (Fig. 3)	Grey Foam Pipe Insulation ID: 15 mm Thickness: 20 mm	4 m	£2.50	£9.99	https://www.ebay.co.uk/itm/Grey-Foam-Pipe-Insulation-Tube-Lagging-Wrap-Roll-Copper-Pipe-Lag/163321870120?hash=item2606bdf728:m:mwI8QGkb7QmoNL_mpC28RYg	Polyethene
Cooling Platform (Fig. 3)	A3 Clear Acrylic Perspex Sheet	1	£6.90	£6.90	https://www.ebay.co.uk/itm/297-x-420mm-A3-Clear-Acrylic-Perspex-Sheet-Plastic-Plexiglass-Panels-2mm-10mm/302379162124?hash=item466733da0c:m:mXb7fyEvSE6X5Z5dc8ZOWPw	Acrylic
Cooling Platform (Fig. 3)	M3x40mm Allen Bolt	3	£0.38	£1.89 (5 pcs)	https://www.ebay.co.uk/itm/M3-M4-M5-M6-M8-M10-DIN-912-HIGH-TENSILE-CAP-HEAD-ALLEN-BOLTS-BLACK-SOCKET-SCREWS/191631586181?hash=item2c9e21f385:m:mgHPnAAtMgQYYslnUgRPuYw	Stainless Steel
Cooling Platform (Fig. 3)	M3 Stainless Steel Nut	3	£0.10	£0.99 (10 pcs)	https://www.ebay.co.uk/itm/M2-M2-5-M3-M4-M5-M6-M8-M10-M12-STAINLESS-STEEL-A4-MARINE-GRADE-FULL-NUTS-NUT-BW/111574104918?hash=item19fa55bb56:m:mFPepilyaxi-g_6nierh-_w	Stainless Steel

## 6 Build instructions

### 6.1 Extrusion system

The extrusion system consists of the components shown and labelled in Fig. 1. Print all the 3D-printing components before starting the assembly. The system can be constructed safely as follows:

1. Cut the plexiglass block (B) in a rectangular form with dimensions 344.4 × 175.0 mm and manufacture the threads based on the CAD file provided.
2. Mount the two linear rails (E) to the printing component (I) through eight M4 bolts.
3. Reduce the length of the M5 bolts from 16 mm to 14 mm.
4. Mount the two nuts (K) to the printing component (I).
5. Connect the lead screw (H) to the assembly through the nuts (K).
6. Insert the two shaft rods (G) into the linear rails (E).
7. Mount the two pillow bearings (D) on the edges of the lead screw (H).
8. Mount the four linear rail shaft supporters on the edges of the two shaft rods.
9. Fix the four linear rail shaft supporters (C) and the two pillow bearings over the plexiglass basement through ten M5 bolts. Do not mount yet the two bolts which connect the 3D-printing components (J) with the two linear rail shaft supporters (C).
10. Fix the motor supporter (M) to the plexiglass basement through two M5 bolts.
11. Connect the stepper motor (A) to the lead screw (H) through the coupler (N).
12. Connect the syringes (F) with the 3D-printing components (J). For the 10 ml glass syringes you have to use Syringe_Supporter file while for the 60 ml the Syringe_Supporter_120.
13. Fix the 3D printing components that support the syringes (J) to the plexiglass basement.
14. Connect the silicon tubing to the nozzle of the syringes.

### 6.2 Needle to tubing connection

The silicone tubing from the dual syringe extrusion system is connected to the stainless steel printing needle using a 15 mm length Luer-lock connection procured from cutting the end of a 1 ml Luer-lock syringe (BD Plastipak, USA). The silicone tubing is fitted to the outside of the Luer-lock connection by stretching the material. The Luer-lock male taper fits in the stainless steel printing needle.

### 6.3 Extruder head attachment

The double extrusion needles were attached to the existing Ultimaker XY moving stage via the novel modular extruder head attachment as detailed in Fig. 2. The construction can be safely achieved as follows:

1. 3D print the extrusion system supporter with filename ‘Extruder_Head_Supporter.stl’.
2. From an appropriately sized aluminium block, machine the heating block to the specifications shown in the CAD design file ‘Heating_Block.SLDPRT’.
3. Place the heating element, in the central hole of the heating block, as shown in Fig. 7.

**Fig. 7:**
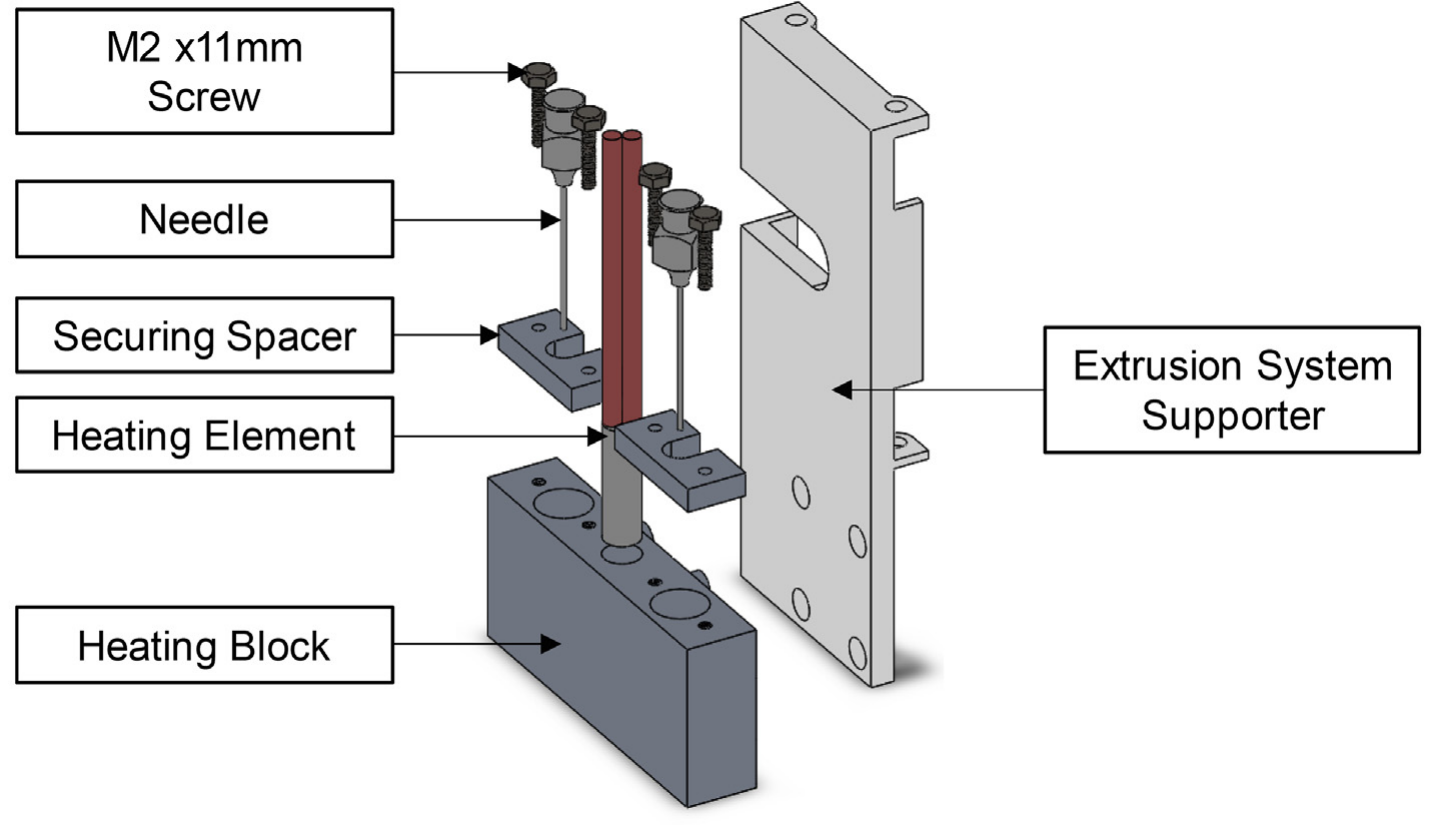
Exploded CAD view of all extruder head attachment components, including optional spacer.
4. Slot in the dual needles to the holes on either side of the heating element, as shown in Fig. 7.
5. The thermistor should be secured to the outside of the heating block, as close to the needle as possible, using adhesive tape.
6. Connect the heating block to the extrusion system supporter.
7. Connect the extrusion system supporter to the Ultimaker XY moving stage. Secure using adhesive tape.
8. Optional: If necessary, the needle can be fixed to the heating block using a spacer block, filename ‘Needle Holder.SLDPRT’ and two M2 × 11 mm screws, as shown in Fig. 7.

### 6.4 Heating system

**Fig. 8:**
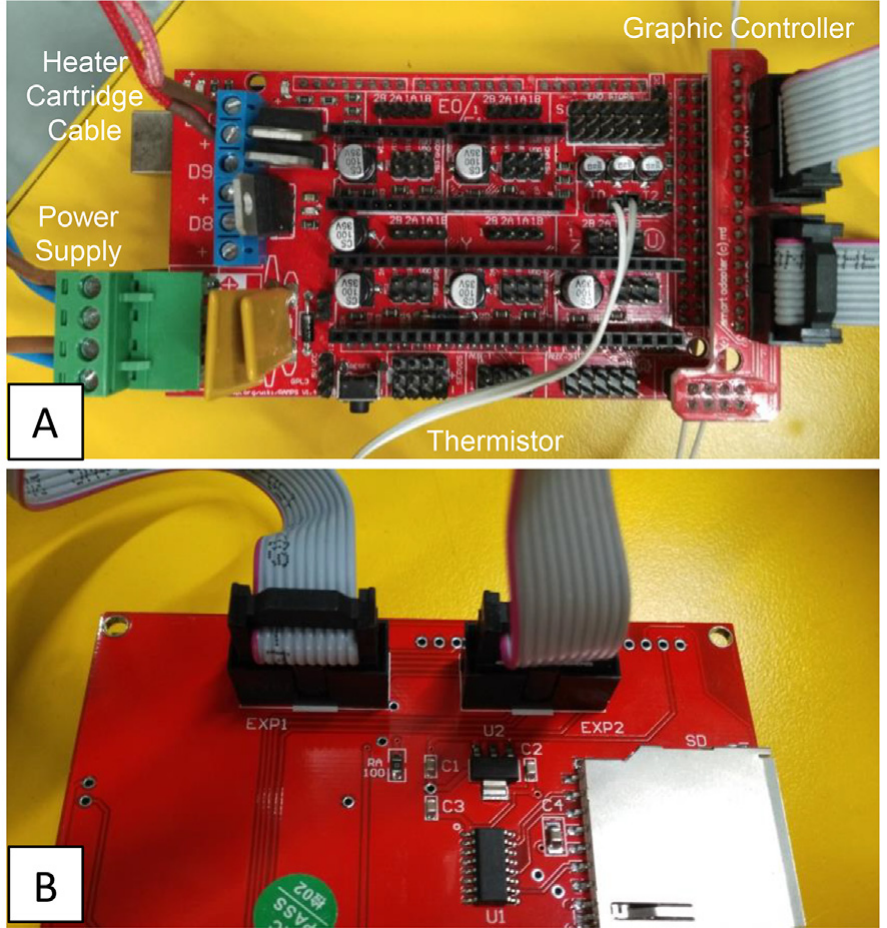
Detailed close up images of the electrical connections for the construction of the heating system where (A) shows the how the components are connected on the board and (B) shows the back side of the interface panel.

The heating system consists of the components shown and labelled in Fig. 6, with the heating components belonging to the extruder head attachment detailed in Fig. 7. Close up images of the Arduino connections are available in Fig. 8. Caution should be used when handling electronic components. Ensure that the power supply is not turned on and connected to the system last. The system can be constructed safely as follows:

1. Connect the leads from the EXP1 and EXP2 ports on the graphic controller to the respective EXP1 and EXP2 ports on the Arduino.
2. Connect the heater cartridge cable to the D10 connection slot on Arduino and screw in the leads to secure the connection. Place the heating end inside the extruder head cavity as detailed in section 5.3 and secure with thermal tape if necessary.
3. Connect the thermistor leads to the T1 slot on the Arduino. Secure the thermistor element to the extruder head as close to the needle as possible to obtain the most relevant measurement of needle temperature.
4. Connect the two positive (blue) and two negative (brown) live wires of the Arduino to the positive and negative ports of the power supply.
5. Check all connections are secure.
6. Plug the table top power supply into the mains.
7. Turn the voltage to 12 V.
8. Use the graphic controller to set the desired temperature.

### 6.5 Cooling platform

The cooling system was connected to the printing platform according to the design described in Section 2.3. The construction can be achieved as follows:

1. Arrange the 4 heat sink radiators so that all the nozzles are facing the same direction, leaving a 10 mm space in between each radiator.
2. Cut pieces from a sheet of polypropylene that it fill in the space in between the radiators and extends to the outer edge of the build plate, leaving space for the build plate corner screws, as shown in Fig. 9(A). Separate pieces may be necessary.

**Fig. 9:**
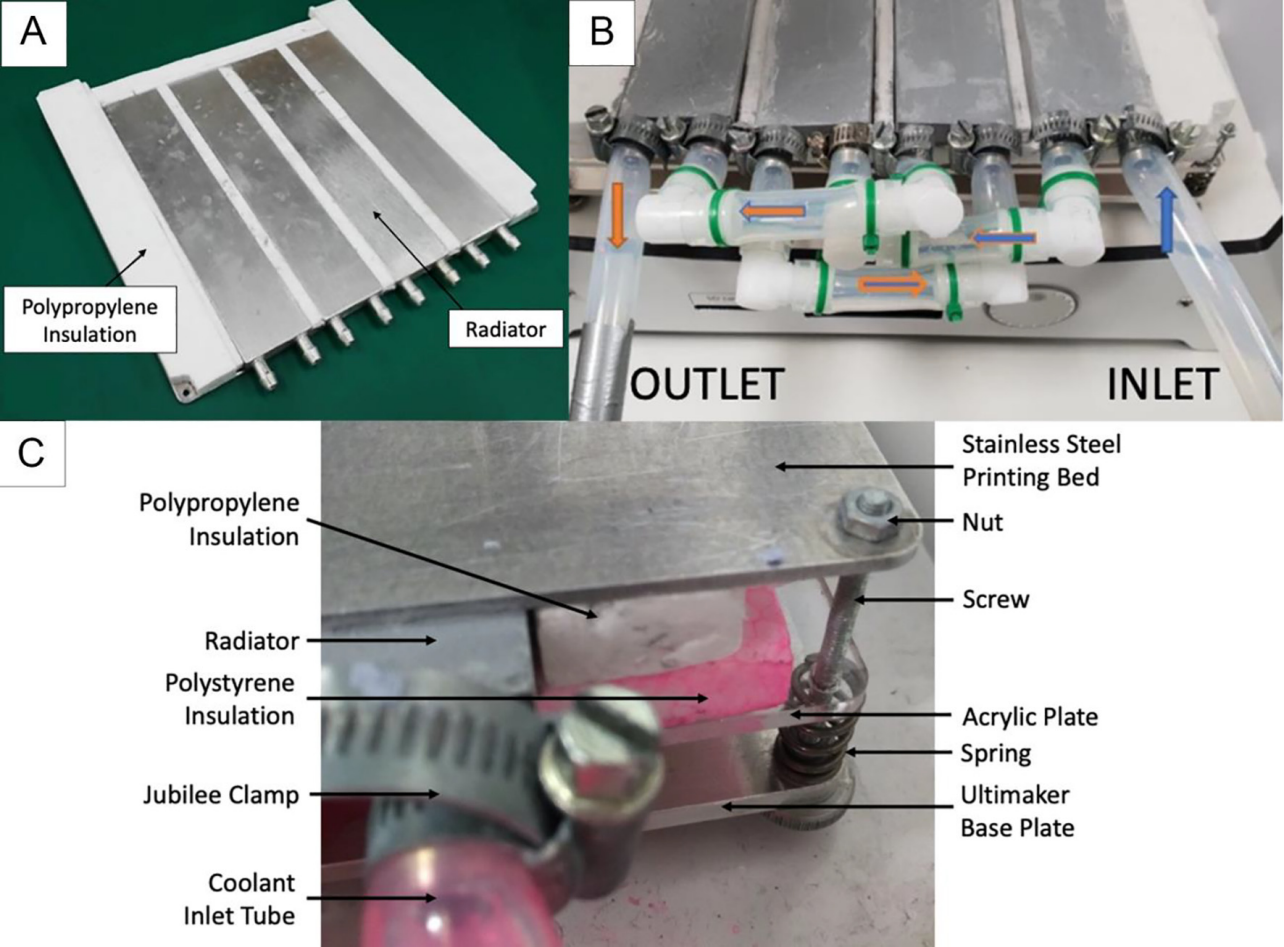
(A) Assembly of heat sink radiators encased in polypropylene, (B) cooling system construction details showing tubing configuration indicating the flow of the coolant and (C) detailed diagram of construction of build plate layers.
3. Place the radiators on top of a polystyrene sheet that has the same area dimensions of the Ultimaker^2^ build plate and also leaves space in the corners for the build plate screws. Arrange the polypropylene pieces around the radiators and use thermal glue to adhere the parts together.
4. Connect the PETG tubing to the 4 heat sink radiator nozzles. Use the Jubilee type stainless steel hose clamps clips to secure the tubing to the component and tighten as much as possible.
5. Secure the connections between PETG tubing and the barbed elbow tubing connectors in the configuration detailed in Fig. 9(B) using plastic cable ties.
6. Place the construction so far on a 3 mm thick acrylic plate with the same dimensions as the build plate area. Laser cut a 5 mm diameter hole in each corner of the acrylic plate.
7. Assemble the screws in the corners of the Ultimaker base plate as shown in Fig. 9(C). The screw is inserted first from the bottom and upwards through the base plate, then the original Ultimaker spring is inserted through the screw on top of the base plate.
8. Place the acrylic plate and cooling system assembly onto the screws.
9. Place the stainless steel printing bed on top of the radiator assembly, ensuring that it is in contact with the radiators for direct heat conduction.
10. Secure the entire assembly in place using a nut through the base plate screw, on top of the stainless steel printing bed.
11. Connect one side of the tubing to the VWR Scientific bath/circulator outlet and the other tube to the outlet. Secure again using the Jubilee type stainless steel hose clamps clips.
12. Wrap grey foam pipe insulation around the inlet and outlet PETG tubing connecting the cooling platform to the VWR Scientific bath/circulator.
13. Ensure the VWR Scientific bath/circulator radiator fans are not blocked to prevent overheating and improve thermal sink efficiency.
14. Turn pump speed to ‘HIGH’ as shown in Fig. 10.

**Fig. 10:**
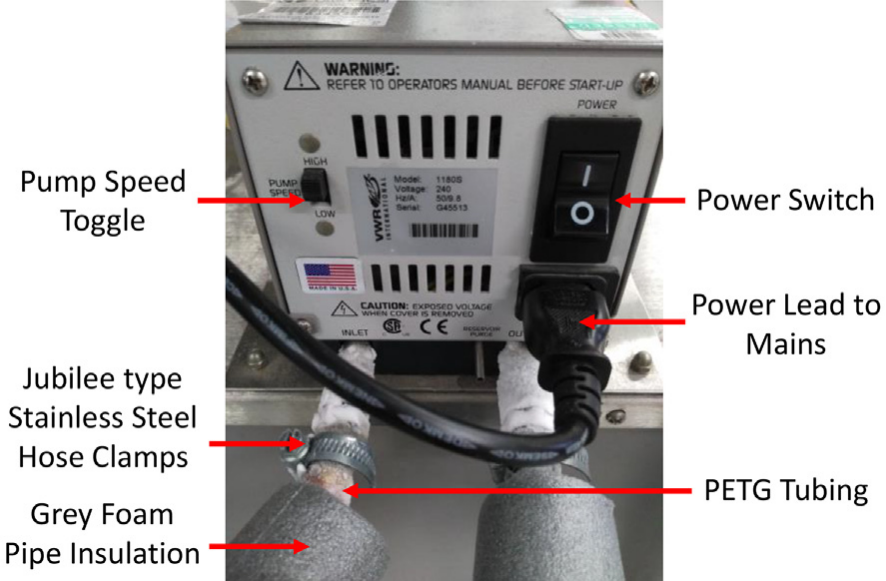
Operation of the VWR Scientific bath/circulator.
15. Plug the VWR Scientific bath/circulator into the mains.
16. Turn on the power switch to start pumping the coolant through the tubing and around the system.

### 6.6 Thermally insulated chamber

The printing chamber was thermally insulated to improve cooling efficiency using multiple layers of 23 mm thick polystyrene sheets to encapsulate the 3D printer and an acrylic door, as described in Section 2.4. The insulation was constructed as follows:

1. Cut 4 400 × 400 mm polystyrene sheets to make the inner layer and 4 423 × 423 mm sheets for the outer insulating layer using a knife tool, for example a box cutter, taking care not to cut fingers and wearing safety gloves.
2. Assemble the insulation around the 3D printer by placing the one inner polystyrene sheet under the printer. Secure the other 3 inner polystyrene sheets to the base sheet using a sealant, e.g. silicone sealant. Use the sealant to seal the side sheet pieces together.
3. Repeat the process with the outer polystyrene sheet layer.
4. For the front of the printer, cut a polystyrene sheet that frames the front space by removing a 300 × 300 mm area from the centre of the piece. Seal the sides and bottom of the frame to the other polystyrene pieces.
5. Optional: to speed up the initial cooling process, an additional removable polystyrene lid can be placed on top of the print, thus fully insulating all 6 sides of the printing chamber. However, the lid must be removed during printing so that the print head is free to move without obstruction.
6. To construct the front door flap: laser cut a 300 × 300 mm piece of 3 mm thick perspex with two 20 × 200 mm slits that allow the inlet and outlet coolant tubing to move unobstructed when the build plate moves during printing, as shown in Fig. 11. Also, laser cut 4 5 mm diameter holes in the perspex flap so that it can be secured to the metal hinge piece, in the positions shown in Fig. 11.

**Fig. 11:**
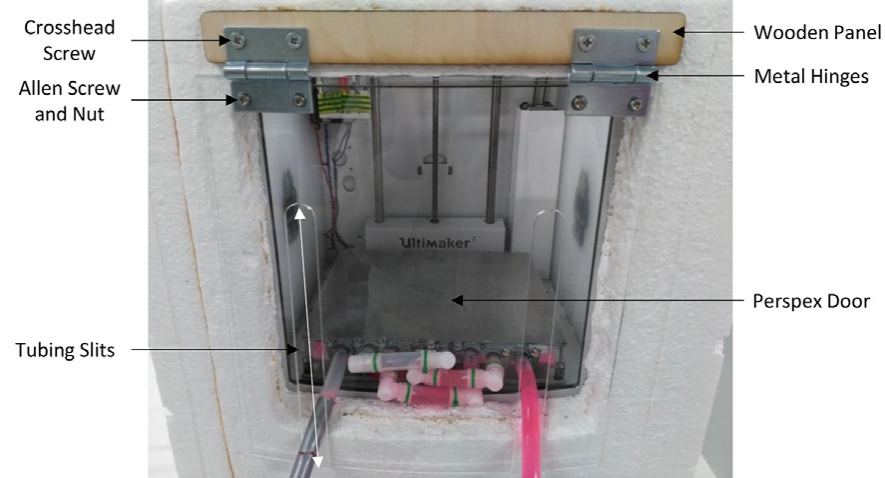
Labelled diagram of 3D printer front door flap.
7. Cut a 300 × 30 mm piece of 5 mm thick wood. Use an adhesive glue to secure the wooden panel to the front polystyrene panel of the insulating chamber to provide support for the door hinges.
8. Attach the metal hinges to the wooden panel using 4 M5 crosshead screws.
9. Attach the perspex piece to the metal hinges using 4 M5 allen screws and nut counterparts.
10. Allow the perspex flap to rest against the polystyrene insulation. The flap can be lifted to clean and maintain the printing plate.

## 7 Operation instructions

1. Turn on power for coolant bath and circulatory system. System will take around 3 hrs to reach lowest temperature. Do not touch the liquid coolant.
2. During this time, check that the tubing and needle system is flowing freely. Identify and clean out any blockages. Connect the needle to the luer-lock tubing and secure it in the extruder head.
3. Turn on power of benchtop power supply for the heating system and set voltage to 12 V.
4. Turn on power for Ultimaker.
5. Calibrate build plate height using Ultimaker controller wheel, as indicated in Fig. 12, to navigate the LED menu. Go to ‘MAINTENANCE’ -> ‘BUILD PLATE’. Turn controller wheel until the needle is around 0.3 mm distance away from build plate. Press into the wheel to confirm and finish calibration. The build plate and nozzle will return to home position.

**Fig. 12:**
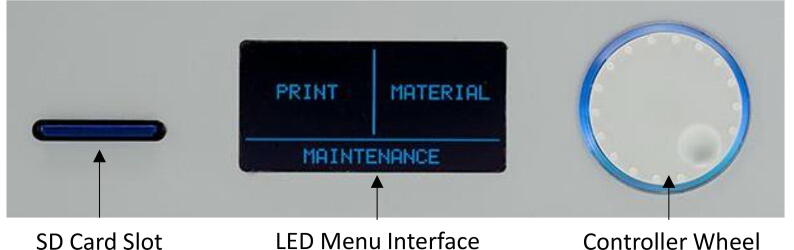
Labelled diagram of the Ultimaker^2^ control panel.
6. Insert SD card with .gcode file of desired printed structure into the SD card reader. Use controller wheel to locate file by going into ‘PRINT’-> ‘filename.gcode’. Press into the wheel to start printing the part.
7. All advanced settings, including the implementation of dual nozzles should be laid out in the .gcode file. Nothing further needs to be done during operation of the modified 3D printer.
8. Throughout operation, for safety purposes, do not put hands inside the printing chamber while the printer is active and avoid spillage and contact with liquid coolant.
9. To stop a print midway, use the controller wheel to select ‘TUNE’ -> ‘ABORT’.
10. To turn off the system after printing is finished, turn off power to coolant bath and circulatory system, benchtop power supply and Ultimaker.
11. Remove the needle from the extruder head and then from the luer-lock tubing. Thoroughly clean the needle by passing hot water through the needle from a 10 ml syringe. Dry the needle by passing air through from a 10 ml syringe. It is best to store needle separately to avoid blockages.

## 8 Validation and characterization

The achievable accuracy of the 3D printer was tested by printing complex-shaped objects [33], [34]. These objects have sharp edges and their construction is challenging because of the hydrogel’s low-stiffness and the over-deposition on the corners due to the inertia of the fluid and the velocity change.

**Fig. 13:**
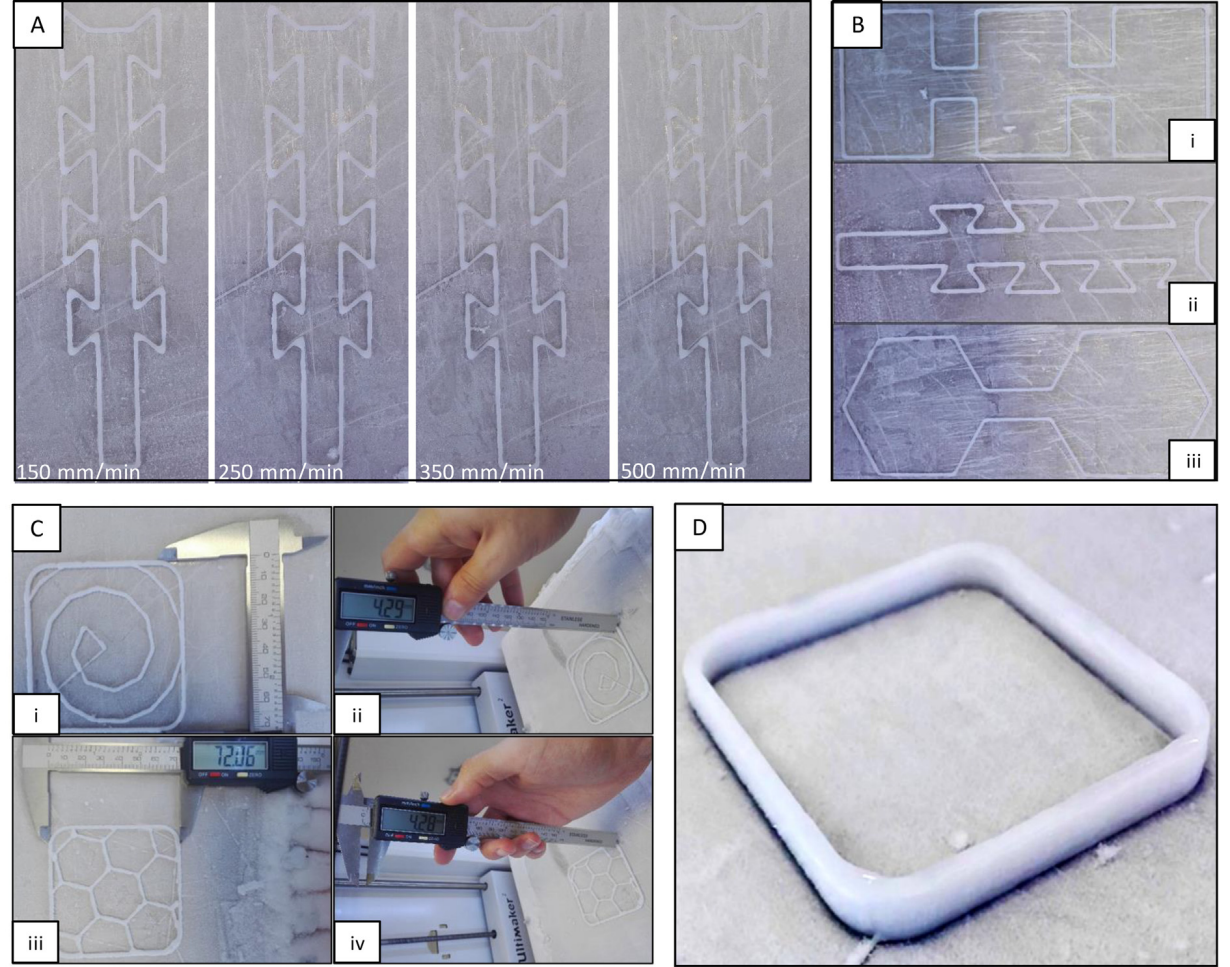
(A) Same object printed at different speeds, (B) printed object with (i) right angles (ii) sharp angles (iii) obtuse angles, (C) (i-ii) Archimedean chord infill (iii-iv) Honeycomb infill and (D) object with 15 layers, 9.4 mm, in the z direction.

Therefore, an object with sharp edges was printed multiple times by keeping all the parameters constant except for the printing velocity (see Fig. 13 A). The line width was almost uniform, except at the corners. Overall, it was observed that as the printing speed increased, the quality of the printing reduced. Printing at slower speeds also had another benefit: the longer cooling time allowed the last layer to fully freeze and solidify, before the deposition of the next layer. However, this lead to longer construction times. Therefore, as a trade-off, the suggested optimum velocity is 250 mm/min.

Additionally, the accuracy of the 3D printer was tested by printing two objects with right and obtuse angles, see Fig. 13(B). Based on visual inspection, the results for both items were deemed to be acceptable. The width and height of each layer were uniform, and the over-extrusion was slightly presented at the corners. For the obtuse object, over-extrusion was not exhibited.

To quantify the accuracy of the 3D printer, the dimensional percentage errors of the print’s geometry were calculated. The ideal total height and wall thickness are given by [38]:

$$H_{Total}=H_{FirstLayer}+{(n_{z}-1)∙H}_{NextLayers}$$

$$W_{Total}={W_{layer}∙n}_{y}$$

The layer height settings that produced the highest print quality were as follows, the first layer height was set at 0.3 mm and the layer height step was set at 0.65 mm. It was observed that a lower nozzle height resulted in collisions with previous layers and a higher nozzle height lead to droplets and hence an uneven layer formation. The extrusion width was 0.5 mm. The layer height was slightly bigger than the diameter of the nozzle due to the die swelling in AM [28] the thermal expansion of the solution, which is 95% water, because of low temperatures and condensation [29]. The symbols $n_{z}$ and $n_{y}$ represent the number of the layers in the z and y direction, respectively. As an example, the ideal dimensions of an object of fifteen layers in the z-direction and three layers at the perimeter are: $H_{Total}=9.4mm$ and $W_{Total}=1.5mm.$

The printer was able to achieve complex geometries with a hydrogel printing ink. The first printed pattern was the Archimedean chords, see Fig. 13(C) (i-ii). In this case, the percentage error in the y and z directions were approximately 0.1% and 2% respectively. The second infill pattern had a honeycomb shape with accuracy in the y and z directions that were approximately 0.1% and 1.90%, respectively, Fig. 13(C) (iii-iv). For both objects, the infill density was set to 3% and the number of layers in z-direction was 7.

The 3D printer was capable of printing objects consisting of more than fifteen layers, 9.4 mm, in the z-direction by using only the cooling platform. Fig. 13(D) shows an object with fifteen layers, 9.4 mm, in the z-direction and three layers at the perimeter. The percentage error in z-direction was only 1%. The percentage error of the wall thickness was higher (10.67%). The discrepancies may be due to condensation, which creates ice crystals around the printed object, and consequently increases the wall thickness. Overall, the accuracy of the new 3D bioprinter system is deemed to be acceptable.

Regarding the extrusion system, the system is easy to set up, precise and adjustable, while the extrusion time can be over 49 h without any refilling. The extruder can be heated up to 260 °C allowing the user to select the appropriate printing temperature for achieving the best quality. Furthermore, the heating block protects the needle from blockage which might occur due to the low working temperatures. Additionally, the extrusion rate is variable during the printing, improving the quality at the corners. The new cooling platform can provide uniform temperature over the printing surface.

The new software allows complicated parts, with infill patterns, to be built easily by designing the object in CAD software (e.g. SolidWorks, CATIA V5, etc.), saving the file in a digital form, generating the G code through Slic3r software and uploading the G code on the 3D printer through an SD card. The printing parameters can be defined and tweaked, and the G code program file can be generated easily and quickly. Additionally, the slicing software allows the utilization of support material leading to the printing of more complex objects with a hollow structure.

**Fig. 14:**
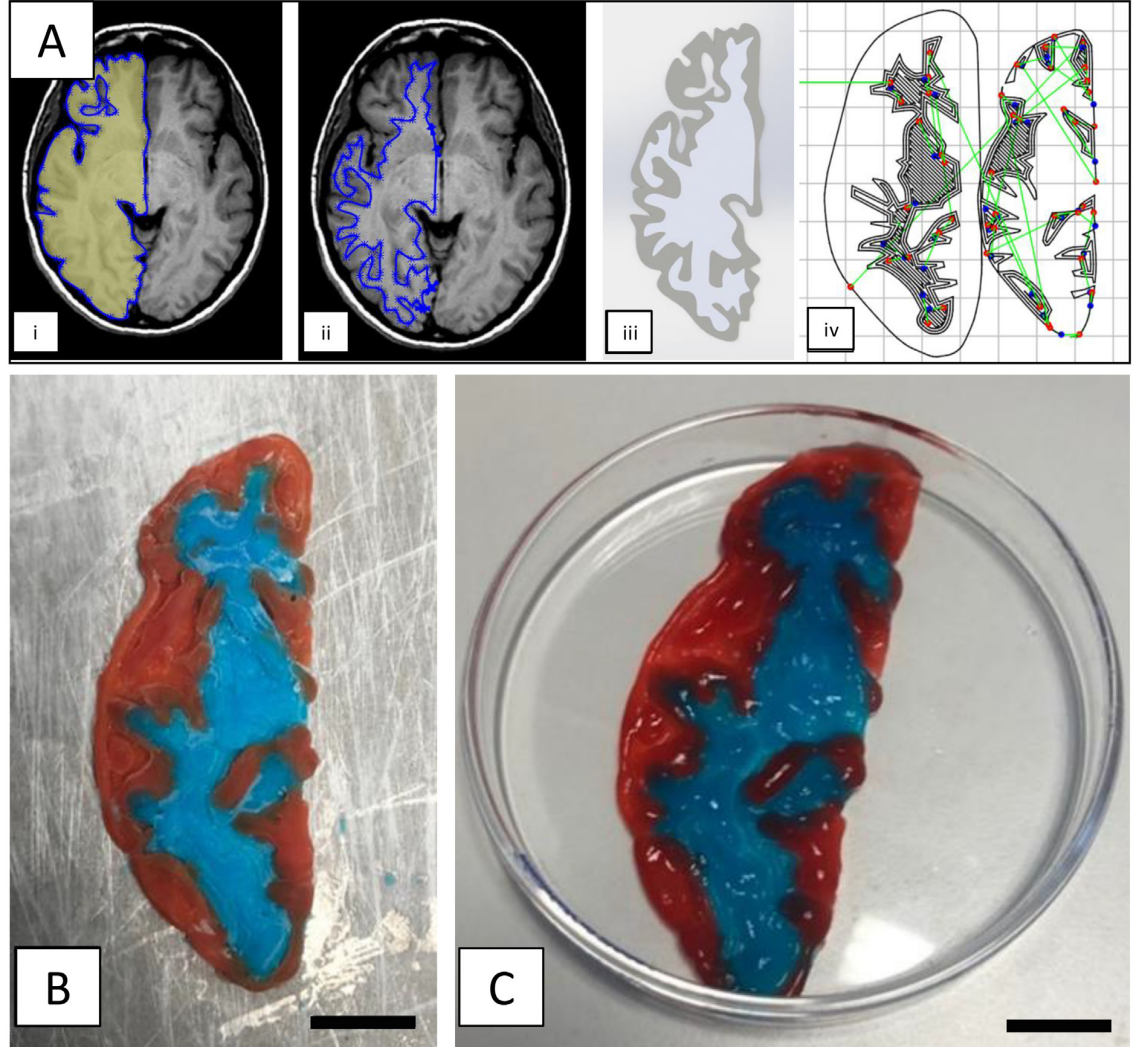
(A) Preparation of 3D printed brain slice from MRI. (i) outlined bitmap trace of the left hemisphere of a human brain MRI [31]; (ii) outlined bitmap trace of white matter areas in the left hemisphere; (iii) output STLs of white and grey matter regions and (iv) extruder path obtained using Slic3r and MATLAB post-processing, and (B) 3D printed brain slice with detailed grey and white matter geometry represented in red and blue, respectively, still frozen just after print completion and (C) final 3D printed brain slice thawed in petri dish after removal from 3D printing platform. Scale bar: (B-C) 10 mm. (For interpretation of the references to colour in this figure legend, the reader is referred to the web version of this article.)

Due to the dual-nozzle modification, the system also enables the fabrication of composite matrices with two different hydrogel compositions that have different mechanical properties. For example, cell growth directionality and migration might be controlled by printing areas with different hydrogel compositions to create a substrate with spatially varying mechanical properties [39]. Complex geometries are also achievable, as shown in the example in Fig. 14. Here, a dual hydrogel printing procedure was employed. The geometry is obtained by an MRI scan of a healthy subject [40] Fig. 14(A) (i-ii). Image processing tools in SolidWorks, including enhanced contrast and bitmap vectorization were used to produce the STL in Fig. 14(A) (iii).

The STLs were translated to GCODE using Slic3r. The code controlling the second extruder was programmed with an offset in the printer X-direction of 35 mm, an extrusion multiplier of 0.1 and a printing speed of 180 mm/min. The extruder paths of a single layer are shown in Fig. 14(A) (iv). A MATLAB code was used for post-processing of the GCODE to input extruder-lift protocols on non-extrusion moves. This eliminates brittle fracture of the print as well as alternation of the primary extruder on each layer to reduce extruder changes. Pre-print extrusion paths were input at each extruder change to ensure reliable extrusion on the print surface. The complex geometry, representing a dual hydrogel brain slice, is shown in Fig. 14(B).

The dual hydrogel brain slice print was stable on removal from the build plate, as shown in Fig. 14(C), with strong adhesion between the two hydrogels. Some intersections of the two hydrogels occurred due to the thickness of printed lines, leading to the loss of fine detail in the print. Material build-up occurred due to over-extrusion at sites of increased angularity, particularly in areas of high infill, leading to some variation in thickness across the print after three layers.

In summary, this contribution presents the full and descriptive details on how to economically achieve a dual nozzle extrusion-based 3D bioprinter based on RFP and cryogenic techniques. It has been demonstrated that the machine is able to fabricate complex geometrical structures using two different hydrogel compositions that represent the cortical white and grey matter architecture, which cannot be achieved through cast-moulding. Upon thaw, these cryogels remain intact and viable as tissue scaffolds for a range of tissue engineering investigations. For example, this machine allows for the fabrication of anisotropic tissue scaffolds with dictated directionality for mechanobiology studies. In addition, 3D embedded tumour models can be fabricated using different hydrogels, representing healthy and diseased tissue separately, which would increase the accuracy of *in vitro* cancer models by replicating the real-life boundary conditions of the tumour microenvironment more closely. Overall, this article allows researchers in biologically applicable fields to easily replicate the machine and benefit from being able to 3D print geometrically complex tissue scaffolds made from materials that mimic the mechanical behaviour of biological tissues.

## Declaration of Competing Interest

The authors declare that they have no known competing financial interests or personal relationships that could have appeared to influence the work reported in this paper.
